# GALNT6 Knockdown Inhibits the Proliferation and Migration of Colorectal Cancer Cells and Increases the Sensitivity of Cancer Cells to 5-FU

**DOI:** 10.7150/jca.62332

**Published:** 2021-10-30

**Authors:** Xiangdong Peng, Xueru Chen, Xiuting Zhu, Ling Chen

**Affiliations:** 1 Department of Pharmacy, The Third Xiangya Hospital, Central South University, Changsha, 410013, Hunan, China.; 2 Department of Gynaecology, The Third Xiangya Hospital, Central South University, Changsha, 410013, Hunan, China.; 3 Department of Gastrointestinal surgery, Xiangya hospital, Central south university, Changsha, 410013, Hunan, China.

**Keywords:** GALNT6, colorectal cancer, AKT, drug sensitivity, 5-FU

## Abstract

The incidence of colorectal cancer (CRC) is increasing annually worldwide, highlighting the need for novel therapeutics to be developed. GALNT6 is a member of the N-acetylgalactosyltransferase enzyme family, which exhibits oncogenic functions in several types of cancers, such as breast cancer and ovarian cancer. However, the function of GALNT6 in CRC has not received much attention in recent years and is therefore poorly understood. In this study, the GALNT6 gene was screened using RNA-seq data obtained from The Cancer Genome Atlas (TCGA). RNA-seq data from 50 pairs of matched CRC patients in TCGA were obtained and analyzed, and the protein expression levels of GALNT6 were verified in 10 pairs of clinical samples. These samples showed that GALNT6 was highly expressed in CRC tissues. Functional analysis also showed that GALNT6 knockdown inhibited the proliferation and migration of CRC cells and increased the number of apoptotic cells. Furthermore, GALNT6 knockdown suppressed tumor growth *in vivo*. GALNT6 also regulated the AKT pathway based on ingenuity pathway analysis and western blotting assay. Finally, GALNT6 knockdown was observed to increase the sensitivity of CRC cells to 5-Fluorouracil (5-FU). These results, taken together, show that GALNT6 knockdown inhibits proliferation and migration of CRC cells and increases cellular sensitivity to 5-FU. It is therefore possible that targeting GALNT6 might prove to be an effective avenue for exploration in any attempt to develop new therapies for the treatment of CRC.

## Introduction

At the present time, colorectal cancer (CRC) is one of the most common malignant cancer types encountered worldwide. Globally, it is the third most frequently diagnosed malignant cancer and causes the second highest number of deaths 1, 2. Its incidence rate increases geographically according to the wealth index of any given country. This positive correlation is thought to be linked to the higher prevalence of alcohol consumption, poor diet and obesity in wealthier nations, as well as certain other lifestyle choices associated with the 'developed world' 3, 4. In recent decades, substantial progress has been made in the diagnosis and treatment of CRC. However, patient prognosis remains poor, particularly in cases where the patient exhibits metastatic CRC 5, 6. To develop new targeted therapies and improved treatments, it will be necessary to further enhance our understanding of the molecular mechanisms underlying the occurrence, development, and metastasis of CRC.

Abnormal glycosylation has been shown to play an important role in the occurrence and development of human malignant tumors 7. Alterations in protein glycosylation underlying oncogenic transformations influence cell-to-cell adhesion impairment, cell migration, and lymphohematogenous metastasis 8. The process of glycosylation describes the transfer of sugar chains to the oxygen atoms of the hydroxyl group of the serine, threonine, or hydroxylysine of the polypeptide chain. Glycosylation primarily consists of N-glycosylation and O-glycosylation which are common post-translational protein modifications observed in cancer cells 9. Abnormal O-glycosylation has been reported to promote the development of CRC by directly inducing carcinogenic characteristics in cancer cells 10. Furthermore, N-acetylgalactosyltransferase (GalNAc-Ts) plays a key role in catalyzing the synthesis of O-glycosylation 11. To date, 20 different GalNAc-Ts human genes have been identified 11. GALNT3 regulates the O-glycosylation of MUC1 via the PI3K/AKT pathway in CRC 12. As one of the 20 types of GalNAc-Ts, GALNT6 has been reported to promote the progression of various types of tumors. For example, in the development of human ductal carcinoma *in situ* (DCIS), the high expression of GALNT6 is closely related to the occurrence of abnormal mucin O-glycation. In addition to this, the expression level of GALNT6 seen in breast cancer cells is substantially increased by comparison to benign, or normal breast cells. Furthermore, the selective expression of GALNT6 in the epithelial cells of some breast cancer patients also seems to be related to angiogenesis and invasiveness 13-15. In breast cancer cells, GALNT6 may regulate the occurrence and metastasis of said cancer by upregulating E-cadherin and the cell adhesion molecule β-catenin 16. Unfortunately, studies aimed at exploring the effect of GALNT6 on the occurrence and development of CRC remain limited.

Herein, GALNT6 expression was significantly upregulated in CRC tissues, facilitating investigations into the function of GALNT6 in CRC and the mechanisms by which it performs that function.

## Materials and methods

### Clinical samples

Matched normal tissue and tumor samples from 10 CRC patients were obtained from Xiangya Hospital of Central South University. These samples were further used to detect GALNT6 protein expression. The present study was approved by the Research Ethics Committee of The Third Xiangya Hospital of Central South University. All patients provided informed consent for use of their samples (No: 2017-S259).

### Bioinformatic analyses

There are 461 available CRC samples in The Cancer Genome Atlas (cancergenome.nih.gov/). Considering that the samples were paired and had complete case information, the RNA-seq data of 41 pairs of matched colon cancer samples and 9 pairs of matched rectal cancer samples were downloaded from the TCGA database. After filtering, standardization, and quality control, the data were analyzed to obtain differentially expressed genes through a general linear model as described previously 17, 18. GEPIA website (http://gepia.cancer-pku.cn/), a free analysis tool, provides differential expression analysis, profiling, correlation analysis, patient survival analysis, similarity gene detection, and dimensionality reduction analysis 19. The box plot of GALNT6 RNA expression in COAD was built in the GEPIA website, which included 275 tumor tissue and 349 normal tissues. Additionally, UALCAN website (http://ualcan.path.uab.edu), not only makes the comparison between primary tumor and normal tissue samples, but also makes the comparison of different tumor subgroups as defined by pathological cancer stage, tumor grade, patient race, and other clinicopathologic features 20. The protein expression levels of GALNT6 in colon cancer were obtained by CPTAC analysis in UALCAN. Z-values represent standard deviations from the median across samples for the given cancer type. Log2 Spectral count ratio values from CPTAC were first normalized within each sample profile, then normalized across samples.

### Cell culture

The human CRC cell lines RKO, HCT116, and SW620 were cultured in RPMI-1640 medium (Thermo Fisher Scientific, Inc.) supplemented with 10% FBS (Thermo Fisher Scientific, Inc) and 1% penicillin-streptomycin. All cells were grown in a humidified incubator with 5% CO_2_ at 37 °C.

### Lentiviral infection

For GALNT6 knockdown, the RNA interference target sequence was designed according to the GALNT6 gene template. GV115 lentivirus vector was used, and the sequence of lentivirus vector was Hu6-GALNT6-CMV-EGFP. The RNA interference target of GALNT6 was GTACCTATGATAATCAGAT.

### Reverse transcription-quantitative (RT-qPCR)

Total RNA was isolated using TRIzol^®^ reagent (Invitrogen; Thermo Fisher Scientific, Inc.) and a total of 2 µg RNA was reversed transcribed into cDNA using a PrimeScript™ RT reagent kit instruction (Takara Bio, Inc). Subsequently, quantitative PCR was performed using an SYBR Green PCR kit (Takara Bio, Inc.). Analysis was repeated three times. The sequences of the primers were:

GALNT6, forward: 5'-CTGTTCTCCATAAACCAGTCCTG-3' and reverse: 5'-CGCTGGCAAAGGCATTGAAA-3'; and GAPDH forward: 5'-TGACTTCAACAGCGACACCCA-3' and reverse: 5'-CACCCTGTTGCTGTAGCCAAA-3'. GAPDH was used as the loading control. Analysis of relative gene expression data using real-time quantitative PCR and the 2-ΔΔCq method 21.

### Western blotting

Western blotting was performed to analyze the expression levels of proteins in cells and tissue. The protein samples were extracted from the cells and tissue using RIPA buffer (Thermo Fisher Scientific, Inc.), and a total of 30 µg protein was loaded onto 10% SDS gel, resolved using SDS-PAGE and transferred to PVDF membranes. The membranes were blocked using 5% skimmed milk for 1 h at room temperature and incubated with specific antibodies overnight at 4 °C. The antibodies used were as: GALNT6 (1:1000, ab151329, Abcam), phospho-(p-)Akt (1:1000, #4060, CST), AKT (1:2000, #4060, CST) and GAPDH (1:5000, ab8245, Abcam). After binding to the specific antibody, the membranes were washed with TBST for four times (5 min each). Subsequently, the membranes were incubated with a horseradish peroxidase-conjugated secondary antibody (1:10000) for 1h at room temperature. Finally, the protein signals were visualized using ECL and the signal strength was analyzed using Image Lab V4.0 software. All antibodies were purchased from CST Biological Reagents Co., Ltd. and diluted in TBST.

### MTT assay

The infected cells were seeded in 96-well plates at a density of 5×10^3^ cells/well and cultured in the medium containing 5-fluorouracil (Selleck Chemicals) at concentrations of 0, 1, 5, 10, or 20 µM. After 48 h, each well was incubated with 200 µl RPMI-1640 medium of 20% MTT solution for 4h in the incubator. Then the 150 µl formazan solution (DMSO) was added to 96-well plates and the absorbance values were detected at 490 nm.

### Colony formation assay

The infected cells were seeded in 6-well plates with 1×10^3^ cells/ well, and the medium was replaced every 4 days. After 2 weeks of culture, cell colonies were washed using PBS and stained with 0.1% crystal violet for 1 h at room temperature and Image J V1.8.0 (National Institutes of Health) was used to count the number of colony formation.

### Transwell assay

Corning Transwell inserts (8-μm pore size; Corning, Inc) were used to access cell migration. In total, 2×10^4^ infected cells were added to the upper chambers of the inserts and cultured in 200 µl serum-free medium, while the lower chambers were filled with 500 µl medium supplemented with 10% FBS. After 20 h in the incubator, the plates were removed, washed with PBS twice, and the upper chambers were stained with 1% crystal violet methanol solution for 30 minutes. After the stained surface was washed with PBS, the chamber was placed under a microscope and 5 fields of view were randomly selected for imaging. The number of cells was calculated by Image J.

### Cell cycle and apoptosis analysis

For the cell cycle analysis, propidium iodide (PI) staining was used and the fluorescence intensity of PI was assessed using a flow cytometer (EMD Millipore). Annexin V-APC single staining was used for apoptosis detection. According to the manufacturer's protocol (eBioscience; Thermo Fisher Scientific, Inc.), 1×10^6^ cells were collected, washed with D-Hanks (pH=7.2~7.4) and 1x binding buffer, and stained with 10 µl Annexin V-APC for 10-15 min in a dark room.

### Microarray hybridization

Total RNA from three pairs of samples, negative control group vs. GALNT6 knockdown group, was analyzed using NanoDrop2000 and Agilent 2100, and prepared into aRNA using GeneChip 3'IVT Express kit. aRNA was purified, fragmented, and hybridized using microarray probes (Affymetrix GeneChip Hybridization Wash and Stain Kit). After hybridization, the chip was washed and dyed. Finally, the images and original data were obtained by scanning.

### Tumorigenesis in nude mice

For tumor formation *in vivo*, the infected RKO cells during the logarithmic growth stage were harvested and injected into the bilateral skin underarms of BALB/c nude female mice (age, 4 weeks; weight, 14-16g). A total of ten nude mice took part in the experimental study. All mice were housed in a temperature-controlled environment (24-28 °C), 40-60% humidity, normal atmosphere with a 12-h light/dark cycle, and provided free access to food and water. When the tumor can be observed, the tumor size is measured. The tumor volumes were calculated according to the following formula: Length x width^2^ /2. Experimental data were collected every 4 days. After 23 days, the experimental animals were euthanized by injection of 2% pentobarbital sodium (150 mg/kg), and death was confirmed death by cervical debarking. Tumors were removed from the animals' armpits for weight and volume analyses. The results were analyzed using a paired t-test. The study was approved by the Animal Ethics Committee of Central South University (No: LLSC(LA)2017-032).

### Wound healing assay

The infected cells were plated in 6-well plates and grown to 90% confluence. Subsequently, a 200 µl pipette tip was used to scratch the monolayer of cells perpendicular to the marking lines. The culture plates were imaged under a microscope at 0, 24, and 48 hours.

### Statistical analysis

Statistical analysis was performed using GraphPad version 8 (GraphPad Software, Inc.). The two groups were compared using the two-tailed T-test, and the multiple groups were compared using the one-way analysis of variance. Bonferroni method was used for the post-test. Paired sample data and animal experiment results were analyzed using paired T-test. P<0.05 was considered to indicate a statistically significant and difference. Each experiment was repeated at least three times and data are presented as the mean ± standard deviation.

## Results

### GALNT6 expression is upregulated in CRC

**Figure 1:**
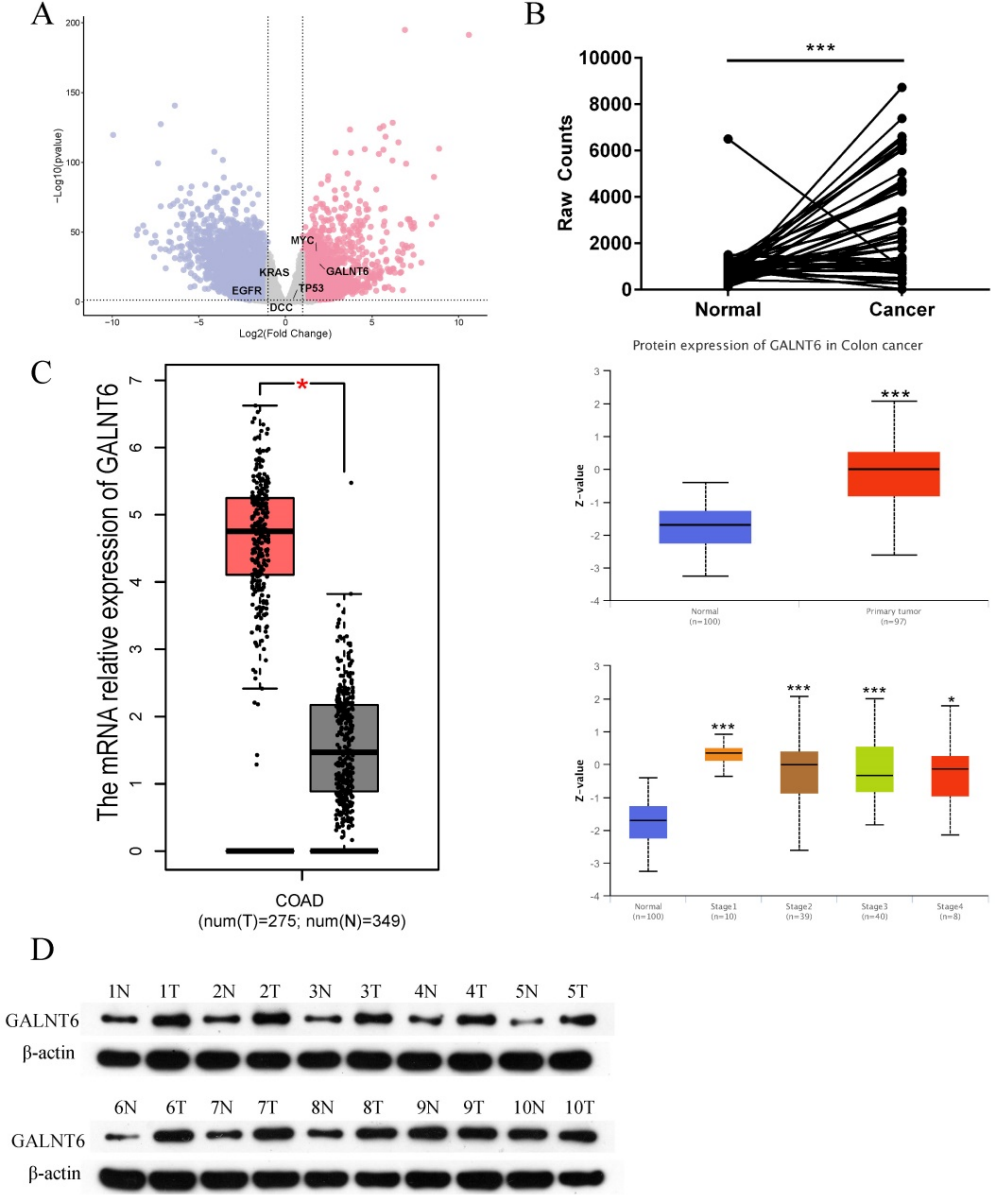
** Expression of GALNT6 in colorectal cancer. (A)** Differential expression analysis of mRNAs between colorectal cancer tissues and adjacent tissues based on data obtained from TCGA. **(B)** Expression levels of GALNT6 in 41 pairs of paired colon cancer samples and 9 pairs of paired rectal cancer samples. **(C)** mRNA expression levels of GALNT6 in COAD based on GEPIA (P<0.05), and the protein expression levels in colon cancer of different stages based on UALCAN analysis (P<0.01). **(D)** Western blotting analysis suggested that the protein expression levels of GALNT6 in 10 pairs of colorectal cancer samples were increased. *P < 0.05, **P < 0.01, ***P < 0.001. All error bars represent mean ± standard deviation.

By analyzing the RNA-seq data of 41 paired colon cancer samples and 9 paired rectal cancer samples from TCGA, numerous differentially expressed genes were identified based on the following threshold of |Log FC|>1 and P< 0.05 (Fig. 1A). To identify the candidate genes, we filtered out the multiple transmembrane protein genes, and unclearly annotated genes, reported in CRC were filtered out. Among the remaining candidate genes, a focus was placed on GALNT6, as GALNT6 was significantly upregulated in tumors (P=5.02×10^-28^, Log FC=3.719). More intuitively, a line chart demonstrated the expression of GALNT6 in 50 paired CRC samples (Fig. 1B). Additionally, the mRNA expression levels of GALNT6 were higher in colon adenocarcinoma tissues with GEPIA analysis. GALNT6 protein expression was further assessed using UALCAN, which showed GALNT6 protein expression was significantly upregulated regardless of cancer stage (Fig. 1C). The protein expression levels of GALNT6 in 10 CRC clinical samples were assessed, and the results demonstrated that GALNT6 was also highly expressed in these cancer tissues (Fig. 1D).

### GALNT6 promotes the proliferation of CRC *in vivo* and *in vitro*

**Figure 2:**
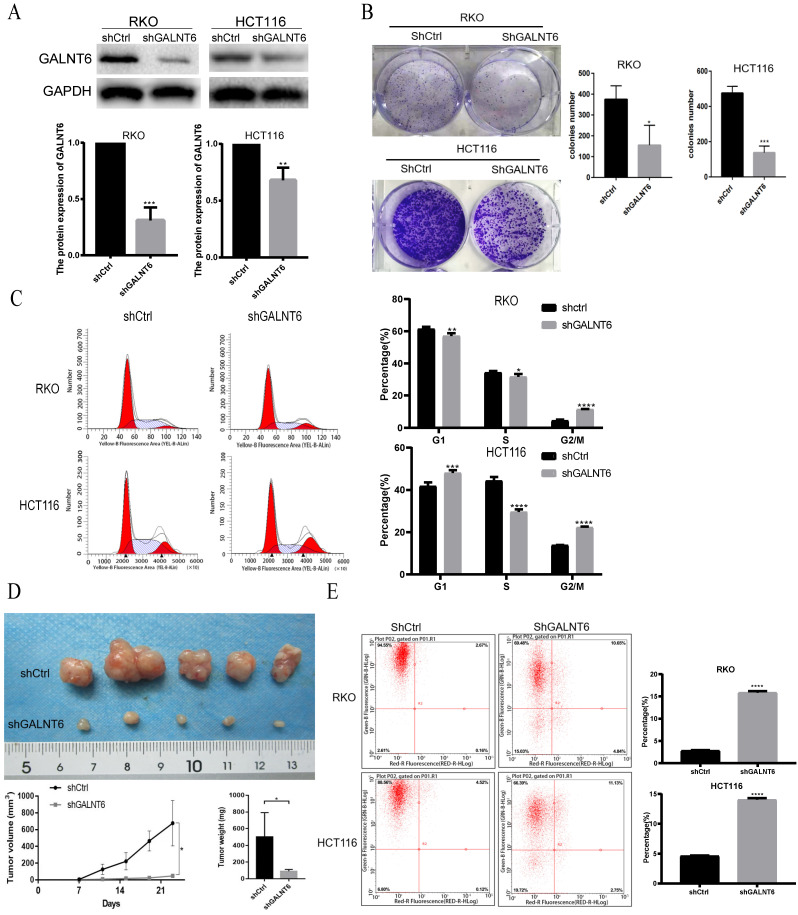
**GALNT6 knockdown inhibits growth and promotes apoptosis of RKO and HCT116 cells. (A)** Results of the western blotting analysis showed that GALNT6 was successfully knocked down in RKO and HCT116 cells. **(B)** GALNT6 knockdown reduced colony formation. **(C)** Cell cycle analysis demonstrated that GALNT6 knockdown mainly increased the proportion of G2/M phase cells in RKO and HCT116 cells. **(D)** GALNT6 knockdown attenuated subcutaneous tumor formation in nude mice. **(E)** Downregulation of GALNT6 increased the apoptosis number of RKO and HCT116 cells. *P < 0.05, **P < 0.01, ***P < 0.001. All error bars represent mean ± standard deviation.

To analyze the detailed functions of GALNT6 in CRC, GALNT6 knockdown cell lines were established. Western blotting was used to verify knockout efficiency, and the results showed that the expression of GALNT6 was significantly decreased (Fig. 2A). Further functional tests showed that GALNT6 knockdown significantly reduced colony formation (Fig. 2B and S1A). Cell cycle analysis showed that GALNT6 knockdown resulted in the significant cell-cycle arrest of RKO and HCT116 cells in the G2/M phase (Fig. 2C), The RKO cells with GALNT6 expression knocked down were used to assess tumorigenesis in nude mice to investigate the role of GALNT6 *in vivo*. The volume and weight of the tumors formed in mice were assessed, and the results showed that both tumor size and volume were significantly decreased in tumors formed of the GALNT6 knockdown cells (Fig. 2D). Furthermore, GALNT6 knockdown also increased apoptosis of RKO and HCT116 cells *in vitro* (Fig. 2E).

### GALNT6 affects the migration of CRC cells

**Figure 3:**
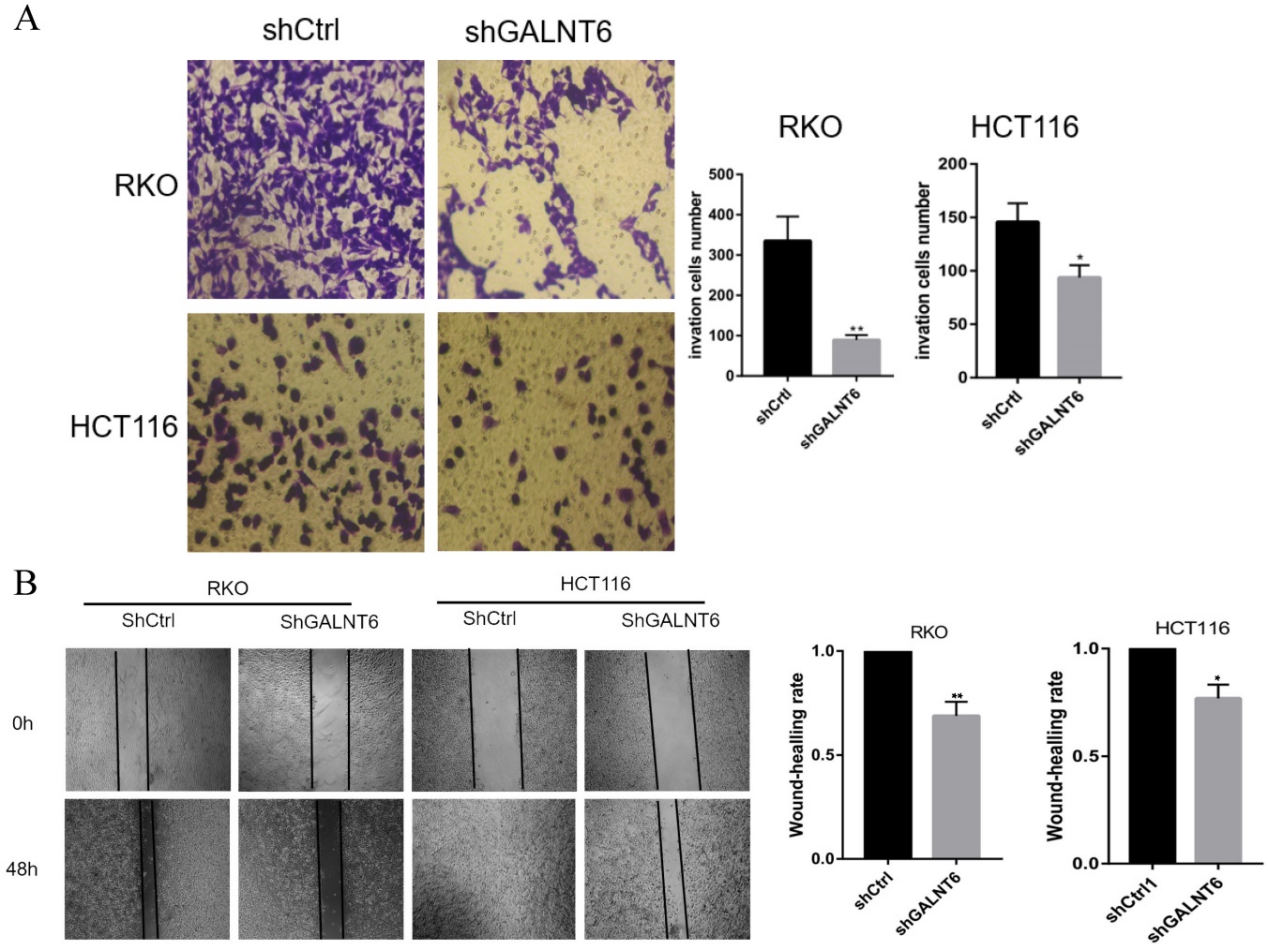
** GALNT6 is involved in the migration of colorectal cancer cells. (A and B)** GALNT6 knockdown reduced the migration of RKO and HCT116 cells in the Transwell migration and wound healing assays. *P < 0.05, **P < 0.01. All error bars represent mean ± standard deviation.

To analyze the effect of GALNT6 on cell migration, the Transwell and wound healing assays were performed. The Transwell assays showed that fewer cells infected with short hairpin-GALNT6 passed through the chamber (Figs. 3A and S1B), and cells with GALNT6 expression knocked down migrated slower (Fig. 3B). These results all showed that GALNT6 knockdown reduced migration in CRC cells.

### GALNT6 regulates the AKT signaling pathway

**Figure 4:**
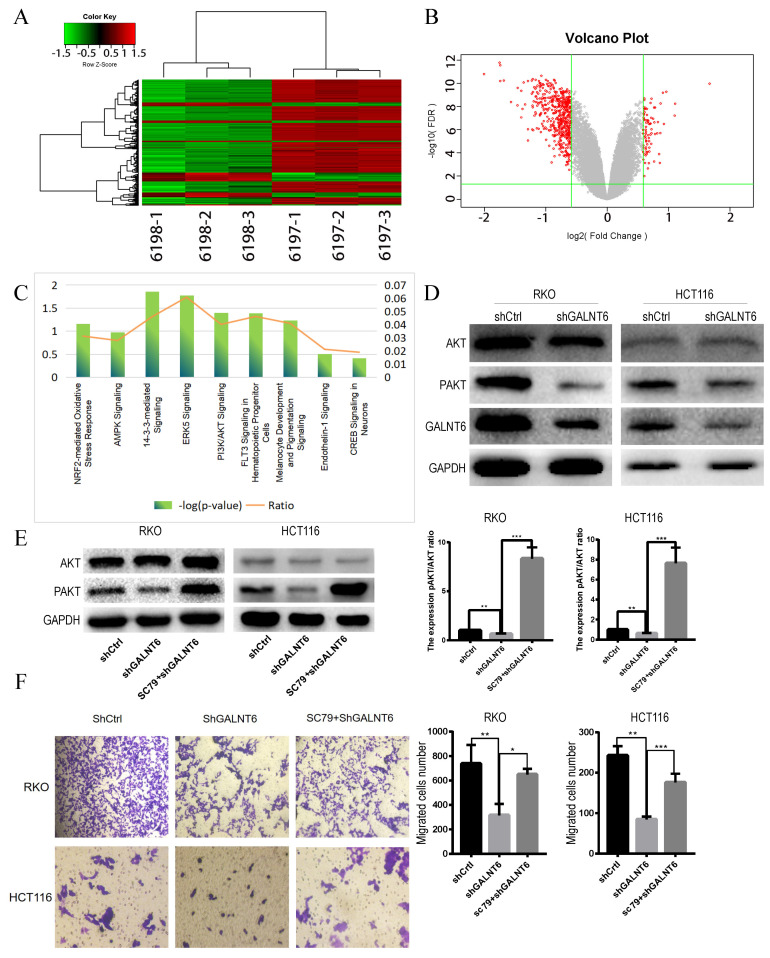
** GALNT6 knockdown reduces the expression phosphorylation of Akt. (A and B).** Heatmap of genes and the volcano plot of differentially expressed genes regulated by GALNT6 in RKO cells. **(C)** The classical pathways affected by GALNT6 knockout based on Ingenuity Pathway Analysis. **(D)** GALNT6 knockdown reduced p-Akt expression in RKO and HCT116 cells. **(E and F)** The addition of SC79 reversed the effects of the GALNT6 knockdown. *P < 0.05, **P < 0.01, ***P < 0.001. All error bars represent mean ± standard deviation.

To determine the biological processes and pathways affected by GALNT6, RNA-seq analysis between GALNT6 knockdown cells and the negative control RKO cells was performed. Based on the threshold criteria (|Log FC|>1.5, false discovery rate <0.05), 355 genes were shown to be affected by GALNT6 knockdown (Fig. 4A and 4B). Ingenuity Pathways Analysis showed that these genes were significantly enriched in 14-3-3-mediated signaling, PI3K/AKT signaling and ERK5 signaling (|Z-score|≥1.5) (Fig. 4C). Based on the literature review and pre-experiment results, we selected PI3K/ Akt Pathway for this study. Western blotting showed that p-Akt expression decreased in the GALNT6 knockdown cells compared with the control cells (Fig. 4D). We used the Akt agonist SC79 to observe changes in protein expression. After the addition of 5 µM SC79 to the knockdown group for 30 min, western blotting showed that SC79 treatment elevated the expression of p-Akt in RKO and HCT116 cells (Fig. 4E). In addition, the migratory ability of the GALNT6 knockdown cells treated with SC79 was enhanced (Fig. 4F). Therefore, it was hypothesized that GALNT6 may affect the migration of CRC cells via the AKT pathway.

### GALNT6 expression is associated with the sensitivity of cells to 5-FU

**Figure 5:**
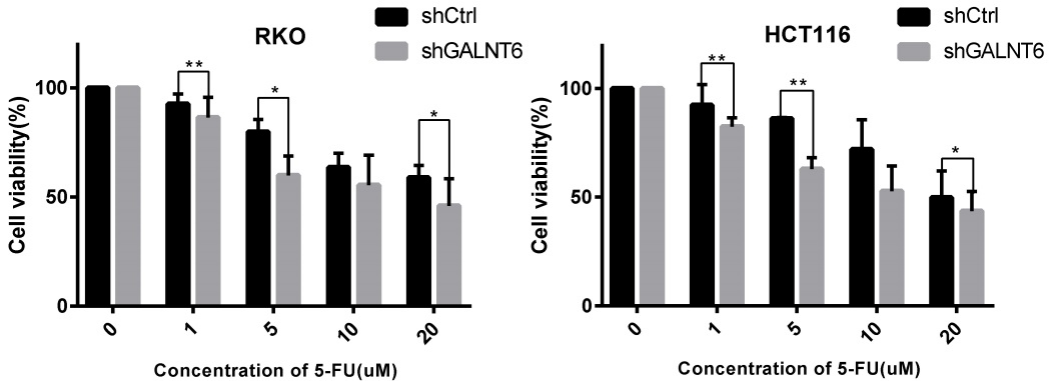
** Sensitivity of colorectal cancer cells to 5-FU.** MTT assay was used to determine the cell viability of RKO and HCT116. *P < 0.05, **P < 0.01. All error bars represent mean ± standard deviation.

To evaluate whether GALNT6 affected the sensitivity of cells to the commonly used therapeutic 5-FU, the cell viability of the GALNT6 knockdown cells and the control group was assessed following treatment with 5-FU. The results showed that the cell viability of the GALNT6 knockdown cells was decreased compared with that of the control group in both RKO and HCT116 cells (Fig. 5).

## Discussion

We investigated the role of GALNT6 in the development and metastasis of CRC. Through the analysis of data obtained from TCGA, as well as from our own clinical samples, GALNT6 expression was found to be upregulated in CRC patients. Additionally, it was shown that GALNT6 knockdown attenuated the formation and proliferation of CRC cells *in vivo* and *in vitro*. Migration was also reduced *in vitro*. Furthermore, GALNT6 knockdown in RKO and HCT116 cells significantly suppressed AKT pathway activity.

Regarding the process of epigenetic change as it occurs within tumors, cell glycosylation is gaining attention as a novel means of studying and understanding the progression of said tumors. Changes in glycosylation not only seem to directly affect cell growth and survival but also appear to promote tumor-induced immune regulation and metastasis 22, 23. Mucin-type, O-linked glycosylation is primarily activated by N-acetylgalactosyltransferase. Abnormal expression of GalNAc-Ts has been observed in several types of tumors. For example, high expression of GALNT1 promotes the acquisition of a malignant phenotype in hepatocellular carcinoma via Epidermal Growth Factor Receptor signaling 24. GALNT6 has recently been identified as an oncogene in several types of tumors. In the case of breast cancer, GALNT6 influences tumorigenicity and metastasis through the β-catenin/MUC1-C pathway 15. GALNT6 knockdown in pancreatic cells results in morphological changes and a cadherin switch from P-cadherin to E-CAD, which plays a crucial role in cell growth and metastasis 13. Dual GALNT3/T6 inhibition is more effective in reducing epithelial ovarian cancer cell proliferation, migration, and invasion, whilst GALNT6 may promote the generation of more aggressive phenotypes via the EGFR pathway 14, 25.

Over the past few decades, 5-FU has shown marked benefits when used as a clinical treatment for CRC. However, in cases where a patient develops a resistance to the treatment, a poor prognosis often follows 26. One area that has seen an expansion of study is the investigation into how and in what proportions 5-FU can be combined with adjuvants to reduce resistance or improve the effectiveness of 5-FU 27, 28. During our study, RKO and HCT116 cells exhibited increased sensitivity to 5-FU following GALNT6 knockdown. Several studies have yielded results to suggest that the development of drug resistance during CRC treatment is primarily associated with genetic and epigenetic changes, such as the acquisition of p53 mutations, overexpression of the anti-apoptotic protein Bcl-2, and Bcl-XL, and alterations in transport-based cellular mechanisms 29-31. Transport-based cellular mechanisms depend on membrane transporters, such as ATP-binding cassette and solute carrier transporters 29. As the mechanisms by which members of the GalNAc-Ts family affect drug sensitivity are not completely understood, any attempts to further explore the mechanisms by which GALNT6 affects the sensitivity of cells to 5-FU would be worth determining.

The extent to which GALNT6 upregulation exerts any effect appears to be dependent on the type of cancer in question. GALNT6 has been shown to attenuate the progression of CRC 16. However, GALNT6 appears to have a different effect on different CRC cell lines. It is, of course, possible that this heterogeneity of effect is due to differences between the cell lines themselves. Phenotypic and functional heterogeneity can occur between cancer cells due to genetic changes, environmental differences, and reversible changes in cellular characteristics 32. The genetic changes observed in the CRC genome appear to be dominated by microsatellite instability which is an important indicator affecting development, chemotherapy efficacy, and prognosis of CRC. Genomic changes within different cell lines can also lead to heterogeneity in the effect of GALNT6 in multiple instances of CRC 33, 34. Studies assessing the function of GALNT6 *in vivo* may therefore be preferable.

In conclusion, the results of our study suggest that GALNT6 knockdown reduces the proliferation and migration of CRC cells through the AKT pathway and enhances the sensitivity of cells to 5-FU. These results highlight the possibility of GALNT6 as a novel therapeutic target for the treatment of CRC.

## Abbreviations

CRC: colorectal cancer
TCGA: The Cancer Genome Atlas
5-FU: 5-fluorauracil
GalNAc-Ts: N-acetylgalactosyltransferase
COAD: colon adenocarcinoma
DCIS: ductal carcinoma *in situ*
RT-qPCR: Reverse transcription-quantitative
PI: propidium iodide
ABC: ATP-binding cassette
SLC: solute carrier
